# The Evolution of the Fœtus Effected by the Action of the Uterus Where the Arm and Funis Presented

**Published:** 1800-01

**Authors:** 


### *The Evolution of the Fœtus effected by the Action of the Uterus where the Arm and Funis prefented.*

ON the 28th of July, 1794, I was called to E. T. aged 26, a well-proportioned woman, and rather under the middle fize. She was in labour of her firft child, and the waters had been difcharged about half an hour. One hand of the fœtus and the funis were protruded beyond the os externum, and the fhoulder was firmly locked in the os internum: the pains, at this time, were ftrong and inceffant; and I found it would be impoffible, without great violence, to turn the child. I returned the funis feveral times into the uterus; but, with every pain, it was forced into the vagina; and I was obliged to fuffer it to remain there. The pains continued unufually ftrong, and frequent, all night, and my attention was wholly taken up in guarding the funis from compreffion; but, notwithftanding all my care, the circulation was frequently checked, though never entirely ftopped. I determined to leave Nature undifturbed, to effect the evolution of the child; for the poffibility of which there was undoubted authority.* Between four and five

* Vide Denman's Introduction to the Practice of Midwifery, p. 294, vol. ii.

five in the morning of the 29th, after twelve hours very hard labour, I was sensible, during the presence of a pain, that the arm was beginning to recede; and, by the power of the next pain, the child was turned, and expelled footling.

